# Role of Extracellular Vesicles Produced by Stem Cells in Tissue Repair

**DOI:** 10.3390/ijms24054798

**Published:** 2023-03-02

**Authors:** Joan Oliva

**Affiliations:** Emmaus Life Sciences, Inc., 21250 Hawthorne Blvd., Suite 800, Torrance, CA 90503, USA; joliva@emmauslifesciences.com; Tel.: +310-214-0065; Fax: 310-214-0075

The purpose of this Special Issue is to emphasize the great potential of the translational applications of extracellular vesicles (EVs) produced by stem cells (mesenchymal stem cells, induced pluripotent stem cells, etc.). Cells release various vesicles, but to avoid confusion, we will be using the word EVs because the majority of the studies did not fully characterize the identity of the EVs (size, protein content, etc.) [1,2]. When studying the potential curative properties of stem cells, due to their low immunogenicity, wide availability, mass production and ability to differentiate into different types of cells [3], researchers have noticed a long-term effect of stem cells on injured tissue, through EVs. Discovered in 1983 [4,5], EVs are biological messengers for cell–cell communication, but the EV content (proteins, lipids, nucleic acids) is specific to the cell type and the cell culture parameters [2,6]. In this Special Issue, five articles studied the effects of extracellular vesicles produced by different types of stem cells (bone marrow stem cells, umbilical cord stem cells, adipose stromal cells, induced pluripotent stem cells) on different organs: human corneal endothelium, mouse cochlea hair cells, rat kidneys, rat cerebral small vessels, human cardiac tissues. Treatment with EVs could overcome various limitations identified in cell therapies: (1) multiple doses can be given to the patient; (2) cell-free therapy with no or a very low risk of ectopic settlement in the patient’s body; (3) no or a low risk of immune rejection.

Knowing the organ’s target and using EVs as a carrying vector, specific miRNAs/mRNAs/proteins could be transfected in isolated EVs or artificial EVs to increase the efficacy of the treatment. Another approach is the modification of the stem cell genome by introducing a specific nucleic acid to produce specific mRNAs, miRNAs or proteins and increase their presence in EVs, in order to enrich their content and improve their curative properties. For example, Buono et al. evaluated the potential of EVs produced by bone marrow stem cells in decreasing endoplasmic reticulum (ER) stress induced by serum deprivation and tunicamycin treatment in freshly collected human corneal epithelial cells [7]. ER stress is well known to be toxic due to the incapacity of the ER to correctly fold the proteins, which accumulate in the cells, and the decrease in ER stress is accompanied by a decrease in cell apoptosis. The authors showed that the miRNAs (miR-222-3p, miR-125b-5p, miR-100-5p, etc.) contained in the vesicles targeted ER proteins involved in ER stress (ATF4, CHOP, etc.). The authors also identified miRNAs and their targets that could be used to improve EV treatment to decrease the harmful effect of ER stress. Remarkably, ER stress is also an activated deleterious pathway in organs with ischemia-reperfusion injury, which occurs during the preservation time of the organ for transplantation. As mentioned, we know that vesicles have a protective effect against inflammation, apoptosis and ER stress, but the factors involved in this mechanism are not clearly identified. The identification of those factors will help to decipher the protective properties of vesicles and also improve their efficacy, as Buono et al. reported [7]. Grignano et al. focused on identifying factors contained in vesicles produced by bone marrow stem cells and used to treat ischemic renal damage. The authors identified CD73, let-7a, miR-21, miR-24 and miR-99a as factors that positively or negatively affected the therapeutic properties of the EVs. One major discovery was that CD73, a surface marker, is involved in increasing the intracellular ATP level (depleted during ischemia) and provides energy to all cellular functions such as protein folding, NADH levels, ATP-dependent transporters, channels and kinase activity [8]. Tsai et al. identified markers in extracellular vesicles produced my umbilical cord mesenchymal stromal cells and used to decrease nerve injury-induced pain in rats [9]. The group identified three major miRs (miR-125a-5p, miR-125b-5p, miR-127-3p) and various proteins (TGF beta ig-h3, collagen alpha-2I chain, collagen alpha 1I chain, glia-derived nexin, etc.) present in the EVs. The authors identified miRs affected by the EVs, compared to the control and disease conditions. Treatment of cochlear tissues with EVs produced by umbilical cord mesenchymal stromal cell exosomes partially restored the expression of 7 miRs out of 40 (miR-181-a-5p, Let-7e-5p, miR-127-3p, miR-183-5p, miR-22-3p, miR-30e-3p, let-7f-5p). Of course, a wider and deeper screening of the affected transcriptome needs to be conducted in further studies, because, thus far, there are at least 2000 microRNAs [10].

In many studies reporting ER stress and inflammation [11], the level of apoptosis decreased after EV treatment. In 2013, the first study to prove the beneficial effect of extracellular vesicles on the treatment of a rat brain after a stroke was reported. Isolated EVs from rat bone marrow were injected into the tail vein of rats to treat stroke. The systemic EV treatment improved the brain activity or protected it against the stroke injuries, but the most remarkable information provided by this study was the potential for the EVs to pass the blood–brain barrier (BBB) [12]. This physical property is a great advantage for EVs over cell therapies because of the limited BBB permeability of activated T cells and not for other cells types as stem cells [13]. Guy et al. studied intranasally administered adipose stromal cell EVs in a rat model that is known to be prone to hypertension [14]. The administered EVs had an impact on inflammation by decreasing pro-inflammatory cytokine production and the BV2 microglial cell and astrocyte activities [14]. In addition, 100% of the rats treated with the EVs survived for 60 days, while only 60% of them survived in the non-treated group. The spatial and visual cognition of the treated rats were superior to those of the untreated rats. In an in vitro study using EVs isolated from human iPSCs, Lozano’s group demonstrated EVs’ protective effect on human cardiomyocytes and human endothelial cells against hypoxia/reoxygenation, a phenomenon that can be encountered during organ ischemia-reperfusion, anemia and congestive heart failure [15,16,17]. The authors fully characterized the proteome of the isolated EVs to better understand what proteins and/or nucleic acids are targeted. The authors proved that the vesicles were absorbed by cardiac fibroblasts and cardiomyocytes. The vesicles protected the cardiomyocytes from apoptosis and improved their capacity to form tubules after a hypoxia treatment. To understand the mechanism of action of the vesicles, the authors identified the pathways and proteins of the cardiomyocytes and cardiac fibroblasts that are influenced by the vesicles, in order to help the cells to self-repair. The majority of the proteins were involved in fatty acyl-CoA biosynthesis, fatty acid metabolism, cholesterol biosynthesis, glucose metabolism and the extracellular matrix [16].

In conclusion, the collection of publications in this Special Issue entitled “Role of Extracellular Vesicles Produced by Stem Cells in Tissue Repair” provides valuable information and knowledge about modified or unmodified extracellular vesicles produced by stem cells. Extracellular vesicles have a wide range of translational applications, including in the treatment of apoptosis, ischemia and endoplasmic reticulum stress. Extracellular vesicles have the advantage of not settling randomly in the body after injection and growth, as with stem cells, but because the research on extracellular vesicles has still not sufficiently advanced, data about the potential risk of organs’ function impairment are still needed in the long term. Major questions concerning the content of extracellular vesicles, which could help to understand their beneficial protective effects, still require additional work before they are answered (Figure 1): How much variability is there among EVs produced by the same type of cells and from different donors? What are the factors influencing the variability in EV contents? How can we standardize EV production specific to the type of disease, organ or injury? What is the optimal combination of EV factors (cell death prevention, increase in cell proliferation, angiogenesis, anti-inflammation, antioxidants, etc.)? Can extracellular vesicles locate the damaged area (e.g., chemoreceptors)? If so, could they be modified to increase their targeting efficiency to the damaged area?

## Figures and Tables

**Figure 1:**
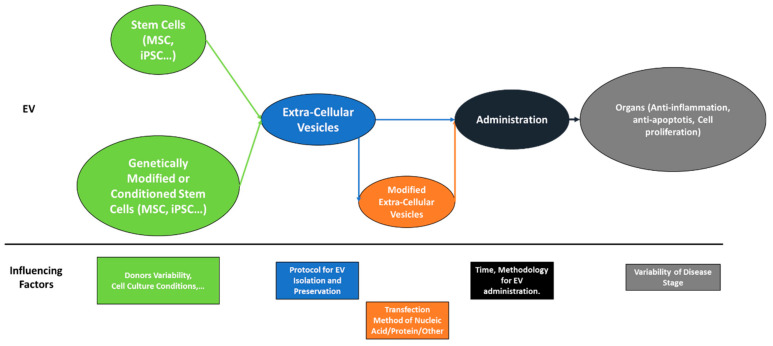
Summary of EVs’ translational applications.
